# Fracture Resistance of Monolithic Zirconia Crowns in Implant Prostheses in Patients with Bruxism

**DOI:** 10.3390/ma12101623

**Published:** 2019-05-17

**Authors:** Ting-Hsun Lan, Chin-Yun Pan, Pao-Hsin Liu, Mitch M. C. Chou

**Affiliations:** 1 Division of Prosthodontics, Department of Dentistry, Kaohsiung Medical University Hospital, Kaohsiung 80708, Taiwan; 2 School of Dentistry, College of Dental Medicine, Kaohsiung Medical University, Kaohsiung 80708, Taiwan; 3 Division of Orthodontics, Department of Dentistry, Kaohsiung Medical University Hospital, Kaohsiung Medical University, Kaohsiung 80708, Taiwan; spig.pan6363@gmail.com; 4 Department of Biomedical Engineering, I-Shou University, Kaohsiung 82442, Taiwan; phliu@isu.edu.tw; 5 Department of Materials & Optoelectronic Science, National Sun Yat-Sen University, Kaohsiung 80424, Taiwan; mitch@faculty.nsysu.edu.tw

**Keywords:** monolithic zirconia crown, thickness, dental implant prosthesis, cyclic loading, finite element analysis

## Abstract

The aim of this study is to determine the minimum required thickness of a monolithic zirconia crown in the mandibular posterior area for patients with bruxism. Forty-nine full zirconia crowns, with seven different occlusal thicknesses of 0.4, 0.5, 0.6, 0.7, 0.8, 0.9, and 1.0 mm, were made by using a computer-aided design/computer-aided manufacturing system (CAD/CAM). Seven crowns in each group were subjected to cyclic loading at 800 N and 5 Hz in a servohydraulic testing machine until fracture or completion of 100,000 cycles. Seven finite element models comprising seven different occlusal thicknesses of 0.4, 0.5, 0.6, 0.7, 0.8, 0.9, and 1.0 mm were simulated using three different loads of vertical 800 N, oblique 10 degrees 800 N, and vertical 800 N + x N torque (x = 10, 50, and 100). The results of cyclic loading tests showed that the fracture resistance of the crown was positively associated with thickness. Specimen breakage differed significantly according to the different thicknesses of the prostheses (*p* < 0.01). Lowest von Mises stress values were determined for prostheses with a minimal thickness of 1.0 mm in different loading directions and with different forces. Zirconia specimens of 1.0 mm thickness had the lowest stress values and high fracture resistance and under 800 N of loading.

## 1. Introduction

Bruxism is a parafunctional activity that includes grinding or clenching masticatory behavior. It might be induced by genetic factor [1,2], central and pathophysiologic, psychosocial, and peripheral factors, or a combination of these [3]. It may occur while awake or asleep with a heavy bite force (BF) and sometimes leads to problematic tooth wear or temporomandibular disorders (TMD) [4,5,6,7]. In awake bruxism, more episodes involve clenching; whereas in sleep bruxism, approximately 90% of episodes involve grinding [8]. Bruxism is always combined with a large BF, which has been taken as a critical indicator of masticatory efficiency. In general, BF is regulated by the muscular, skeletal, nervous, and dental systems. BF might be affected by race, age, sexual dimorphism, the posture of the subject’s head, interocclusal separation, occlusal contact area, and location of the measuring device on the dentition [9]. Bite movement could be classified as voluntary or involuntary. Raadsheer et al. found that the voluntary normal BF was 386.6 N in women and 545.7 N in men. The voluntary maximal BF measured was 576 N in women and 888 N in men [10]. Involuntary bite movement always occurred in sleep bruxism along with tooth wear, root fracture, and prosthesis failure.

An implant-supported fixed partial dental prosthesis is being established as a treatment option for patients who are partially edentulous. After osseointegration, mechanical stresses and strains of hard tissue and prosthesis materials have been considered a critical challenge in the success of implants and implant prostheses [11,12]. Campos et al. [13] published that the points to bear reiteration of clinical materials is affected by materials, failure mode, flaw state, layer thicknesses, and cement. Yuan et al. [14] mentioned that overload from occlusion may cause biomechanical complications of an implant prosthesis, such as bone loss, loss of abutment retention, fracture of ceramic, and dislodging of prosthesis. Sheridan et al. [15] suggested that following the clinical guidelines, such as improving force direction along the long axis of implant, reducing force magnification by increasing prosthesis contact area, and increasing the support area of implant prosthesis, would be helpful in clinical cases. When an implant prosthesis is used for bruxism cases, it would accommodate a large load from clenching or grinding movements. A dentist could figure out the etiology by consulting the patient’s physician and then derive a method of designing either a high load-bearing dental prosthesis or one that uses the stress relief method. Previously, clinicians have always used a full metal prosthesis to help patients with bruxism symptoms [5,6,7]. Ceramic restoration is popular because of its excellent optical properties. However, ceramic restoration failures are an undesired outcome. De Souza Melo et al. [16] stated that the complex relationship between the occurrence of restoration failure and occlusal overload remains unclear.

Yttria-stabilized tetragonal zirconia polycrystal (Y-TZP) has attracted research attention owing to its biocompatibility, mechanical properties, and excellent natural appearance compared with metal restoration, as reported by Piconi and Maccauro; it possesses relatively high flexural strength (1000 MPa), elastic modulus (215 GPa), and fracture toughness (6–10 MPa·M^1/2^) compared with other ceramics [17]. Zhang et al. [18] reported that radial cracking of ceramic flat-layer specimens induced by spherical indenters on their top surfaces is highly correlated to the thickness of the ceramic layer. Hamburger et al. [19] found that the fracture risk of ceramic materials is highly dependent on layer thickness. Deng et al. [20] compared different ceramic materials and revealed that the fully dense zirconia (Y-TZP) with low thickness (0.1–1.0 mm) had relatively higher critical load (100–1000 N) compared to other ceramic materials. Researchers and dental technicians suggest that the minimum thickness of zirconia for natural dentition should be 0.5 mm [21]. Alghazzawi et al. [22] demonstrated the ability of computer-aided design/computer-aided manufacturing (CAD/CAM) zirconia laminate veneers with a 0.3–0.5 mm reduction to withstand higher loads before fracturing compared with glass-ceramic. Lan et al. [23] suggested that an implant prosthesis of monolithic zirconia crown with a thickness of 0.8 mm is recommended to allow errors occurred during operation. Kelly et al. [24] suggested using the finite element method (FEM) and stiffer substrate to solve for stresses as a function of load. Hsueh et al. [25] changed with the thickness ratio between the veneering and framework materials and used the FEM to determine that the location of maximum tensile stress. Nejatidansh et al. [26] showed the promising clinical performance of a zirconia-based single crown on both tooth and implant abutments up to a seven-year follow-up. On the other hand, Zhou et al. [27] found that prostheses in bruxers had a higher failure rate than in patients without bruxism by using a meta-analysis to evaluate the relationship between implant failure and bruxism. One expected benefit of monolithic zirconia crowns would be a decrease in clinical ceramic prosthesis failures [28], especially in patients with insufficient interdental distance; however, articles that present clinical recommendations for bruxism are rare.

The survival rate of implant prostheses is also affected by occlusion. Due to lacking periodontal ligament, researchers suggest avoiding large occlusal tables, excessive premature contacts, and steep cusp inclinations [14]. With maximum intercuspation (MICP), a reasonable clinical approach would be examining no contact when an occlusion record is light, and light contact (30 μm) when an occlusion record is heavy [29]. Furthermore, Hsu et al. [30] reviewed and revealed the guidelines for implant occlusion in patients with a bilateral free end or who are completely edentulous and found that there must be contact in the MICP position regardless of light or maximum intensity. This condition indicates less cushion space and more contact in MICP. Reconciling the large force to lower stress could not only help in adhering to the occlusion guideline but also supplement the cushion with an occlusal stabilization splint, or night guard [31].

Porcelain fused to zirconia has good esthetic appearance but is easy to fracture. Porcelain chipping or delamination might be due to mismatch in thermal expansion coefficient between veneer and zirconia core [32]. Therefore, using a monolithic zirconia crown for a posterior implant prosthesis is expected to become increasingly popular [33]. Patients with bruxism have a choice to select esthetic and high-strength prostheses. However, interdental space, loading direction, and loading type affect the success of the prosthesis. Lan et al [23] revealed that an implant prosthesis of monolithic zirconia crown with a thickness of 0.7 mm could afford the natural BF of 300 N and showed the lowest stress value. The null hypothesis of this study was that the monolithic zirconia specimens with thickness of 0.7 mm could afford the heavy BF and showed the lowest stress one. Therefore, the aim of this study is to determine the minimum required thickness of a monolithic zirconia crown in the posterior region for patients with bruxism.

## 2. Materials and Methods

### 2.1. Specimens Preparation

A monolithic zirconia crown in the molar area was designed to set on an implant abutment. The sample size was estimated by G power analysis; seven test groups were assumed, an effective size of 0.76, the probability of α-error of 0.05, and the power of 0.95. The sample size was thus determined to be seven per group. Forty-nine complete zirconia specimens comprising different occlusal thicknesses of (0.4, 0.5, 0.6, 0.7, 0.8, 0.9, and 1.0 mm) were designed by using the CAD/CAM technique. All specimens were milled by the same open computer numerical control (CNC) system milling machine (ARDENTA CNC MILL, CS100-5A, ARIX, Tainan, Taiwan) by using one commercial Y-TZP zirconia block, brand V (made in Bad Sackingen, Germany) and densely sintered at 1450 °C for 2 h.

### 2.2. Cyclic Loading Test

**Figure 1:**
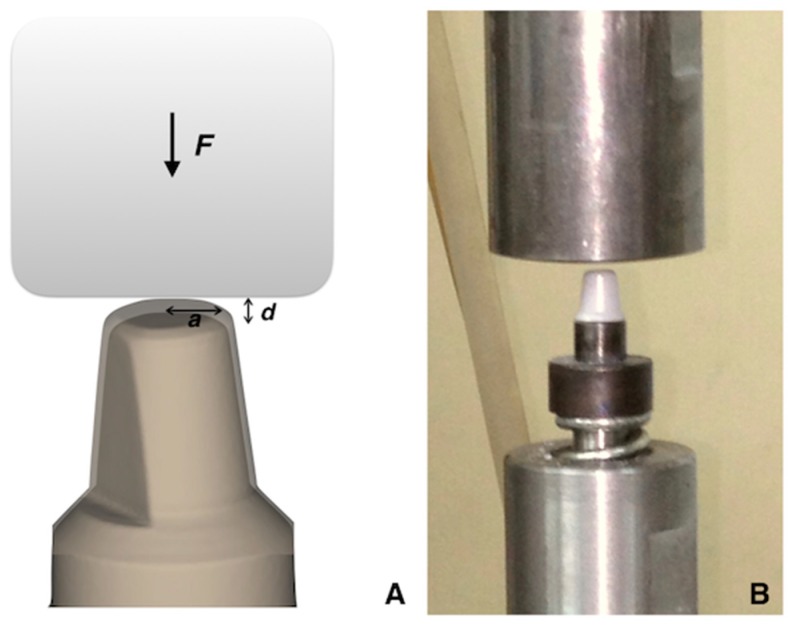
(**A**) Schematic of zirconia specimen. Thickness *d* on implant abutment indented by a cylindrical indenter with a contact area of radius *a* at load *F.* (**B**) Zirconia specimen with holder and indenter to simulate the bruxism movement by the servohydraulic testing machine.

The specimens were set on the implant die without a cement space but with adequate friction retention. Figure 1 shows that the antirotation surface design of the dental implant abutment was helpful to stabilize the prosthesis. Seven specimens in each group were tested vertically with a frequency of 5 Hz and load of 800 N in a servohydraulic testing machine (Instron M8810, Instron Ltd., Norwood, MA, USA) until fracture or completion of automatic stopped after 100,000 cyclic counts. To simulate the cycle of bruxism, the computer program was used to maintain a consistent force during the test cycle. The indentation stress-strain relation for contact with the spheres is well-defined by the classic Hertzian theory for ideally elastic, homogeneous bulk materials using of Young’s modulus *E* and Poisson’s ratio *ν*. The Hertzian solution [34,35,36] has the linear form

(1) $$a=\sqrt{rd}$$

where *a* is the radius of contact area, *r* is the radius of indent, and *d* is the depth.

*P* = 3*F*/2π*a*^2^ (2)

*P* = (4E*/3π)(*a*/*r*) (3)

where *F* is the load, *P* is the maximum contact pressure, and E* is an effective modulus.

E* = 1/[(1 − *ν*_1_^2^)/*E*_1_ + (1 − *ν*_2_^2^)/*E*_2_] (4)

where *E*_1_ and *E*_2_ are Young’s modulus of indenter and specimens, and *ν*_1_ and *ν*_2_ are the Poisson’s ratios associated with each body.

### 2.3. Finite Element Method

Dental implant abutment models were constructed using a CAD software (SolidWorks 2010, SolidWizard Corporation, Concord, MA, USA). Seven FEMs with different occlusal thicknesses of 0.4, 0.5, 0.6, 0.7, 0.8, 0.9, and 1.0 mm were constructed to simulate the posterior molar region. All models were combined through Boolean operations by using CAD software (Pro/ENGINEER Wildfire 2.0; Parametric Technology Corp., Boston, MA, USA). The Young’s modulus and Poisson ratio of Ti-6Al-4V [37] were 110 GPa and 0.33, respectively. The Young’s modulus and Poisson ratio of zirconia [38] were 220 GPa and 0.3, respectively.

The materials used in the models were assumed to be isotropic, homogeneous, and linearly elastic. The 3D FEM of the 0.4 mm thickness abutment consisted of 169,722 elements and 240,446 nodes to mesh by 10-node tetrahedral elements. Four loads, vertical (V) 800 N, V 800 N + torque (T) 10 N, V 800 N + T 50 N, and V 800 N + T 100 N, were applied on the occlusal surface of specimens, and four directions of oblique 10° 800 N loads were applied to the marginal ridge of the specimens. All the directions of the nodes on the mesial and distal borders of the implant-prosthesis complex were constrained by the boundary condition. The maximum von Mises stress values were detected from the specimens in different loading directions.

### 2.4. Statistical Analysis and Microstructural Observation

Kruskal–Wallis test was used to compare data, and statistical program (SPSS Statistics for Windows, v20; IBM Corp., Armonk, NY, USA) was used to determine the Spearman correlation. The microconditions of the specimens were monitored using scanning electron microscope (SEM) imaging (JSM-6360; JEOL, Tokyo, Japan).

## 3. Results

### 3.1. Cyclic Load Test

**Figure 2:**
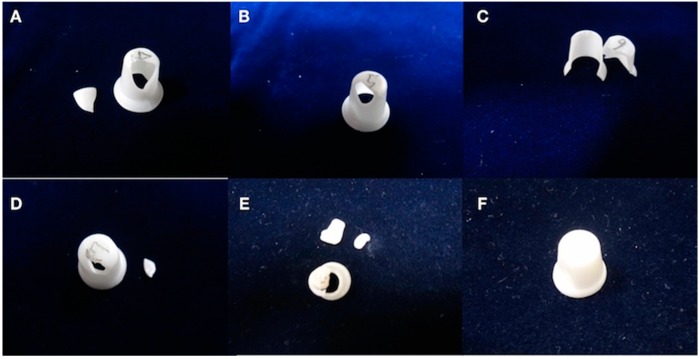
Specimens with equal thickness after testing. (**A**) The 0.4 mm specimen is broken at the corner of the top, and the axial wall has an obviously broken line on the wall. (**B**) The 0.5 mm specimen is broken on the corner of the top and at the axial wall. (**C**) The 0.6 mm specimen has fractured segments. (**D**) The 0.7 mm specimen has two fractured segments. (**E**) The 0.8 mm specimen has three fractured segments, broken in similar areas as the 0.4, 0.5, 0.6, and 0.7 mm specimens. (**F**) The 0.9 mm specimen has an intact morphology. Two of the seven specimens are intact.

Table 1 shows the mean cycle numbers until fracture were 5.4, 12.4, 27.1, 1869.3, 10,346.9, and 50,853.4 for 0.4, 0.5, 0.6, 0.7, 0.8, and 0.9 mm specimens, respectively. Obvious breakages that separated specimens into two to three fragments were noted (Figure 2A–E). Two of seven 0.9-mm specimens (Figure 2F) and all 1.0-mm specimens had no visible fracture lines after 100,000 cycles. Specimen thickness and number of cycles had a strong positive association (r = 0.966, Table 1). Specimen breakage was moderately strongly associated with crown thickness (r = 0.659). Whether a specimen was broken or not was significantly different when comparing thickness and cycle number (*p* < 0.05).

### 3.2. Finite Element Analysis

**Figure 3:**
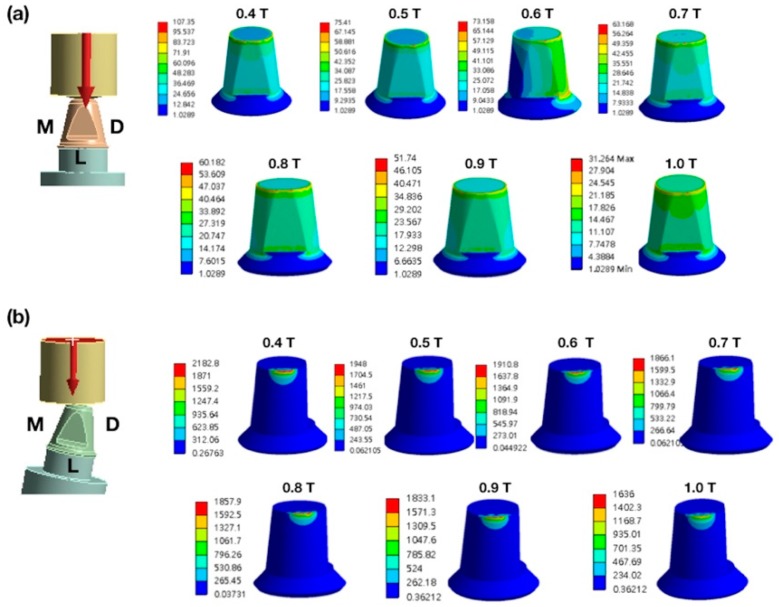
Distribution of stress in zirconia specimens. (**a**) The vertical load of 800 N from the lingual view. (**b**) Oblique 10-degree load of 800 N on the mesial marginal ridge. From left to right are the following loading models: top first row 0.4 T, 0.5 T, 0.6 T, and 0.7 T; top second row 0.8 T, 0.9 T, and 1.0 T; T: thickness (mm). D: distal. M: mesial. L: lingual.

**Figure 4:**
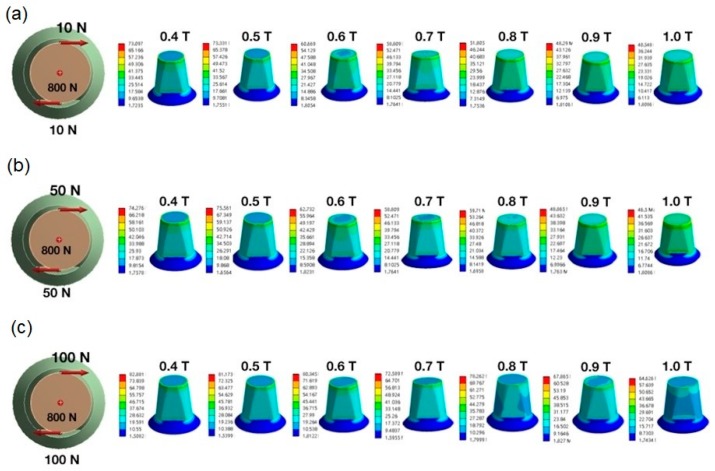
Distribution of stress in zirconia specimens. (**a**) Vertical load of 800 N with torque of 10 N. (**b**) Vertical load of 800 N with torque of 50 N. (**c**) Vertical load of 800 N with torque of 100 N. From left to right are the following loading models: 0.4 T, 0.5 T, 0.6 T, 0.7 T, 0.8 T, 0.9 T, and 1.0 T; T: thickness (mm).

**Figure 5:**
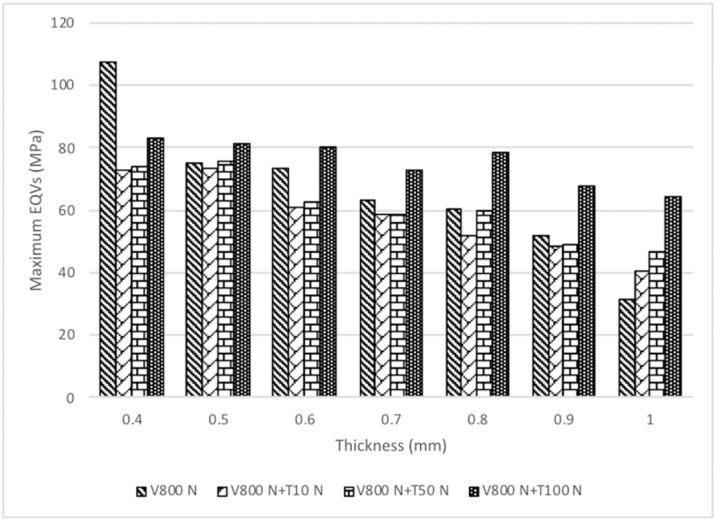
Comparison of von Mises stress values (EQVs) in zirconia specimens with seven thicknesses under vertical (V) 800 N, V 800 N + torque (T) 10 N, V 800 N + T 50 N, and V 800 N + T 100 N. The lowest value occurred when the thickness was 1.0 mm. V: vertical, T: torque.

**Figure 6:**
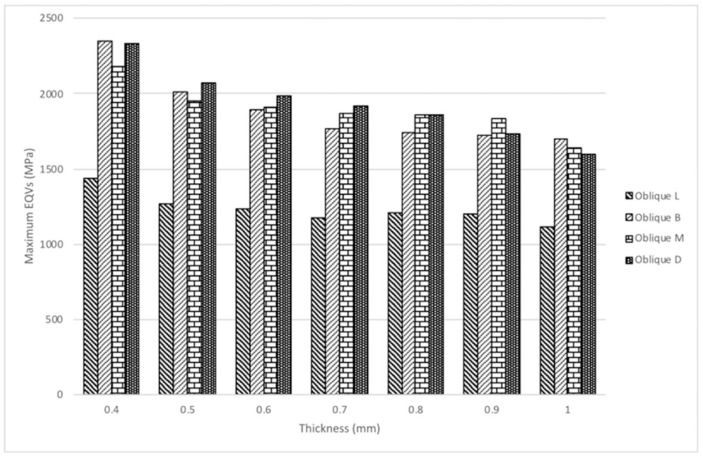
Comparison of EQVs in zirconia specimens with seven thickness under four different oblique 10-degree loading directions with 800 N force. The lowest value occurred when the thickness was 1.0 mm under lingual side loading. L: lingual, B: buccal, M: mesial, D: distal.

Stress induced by forces applied in different directions will concentrate in the corner area of occlusal-axial wall of the specimens. The maximum von Mises stress values (EQVs) were higher with oblique loading compared with other loading types. The peak EQV decreased with increasing thickness under vertical 800-N loading, with 1.0 mm showing the lowest value of 31.26 MPa (Figure 3a). Figure 3b shows the stress distribution of different thicknesses under oblique 10° 800-N loading on the marginal axial area of the specimens, and the 1.0-mm thick specimens had the lowest value at 1636 MPa. Figure 4 portrays the stress distribution of hybrid loading (V 800 N + T 10 N, V 800 N + T 50 N, and V 800 N + T 100 N) with different thicknesses (0.4–1.0 mm). The peak EQVs were concentrated in the corner area of the occlusal–axial wall of the specimens. As long as the torque force increased, the peak stress value increased, and the 1.0 mm specimens had the lowest value. When vertical loading was combined with torque force, greater torque had higher peak EQV, except for the 0.7-mm specimens (Figure 5). Additionally, when the thickness was greater than 0.5 mm, the peak EQV from V 800 N + T 100 N was higher than V 800. When the thickness was 1.0 mm, all of the peak EQVs from the hybrid loading were higher than V 800. Furthermore, with different directions of oblique loading, the peak value from oblique L was lower than those from the other loading directions, and the 1.0-mm thick specimens had the lowest value among the different oblique loading directions (Figure 6).

### 3.3. Microstructure of Zirconia Block after Cyclic Loading Test

**Figure 7:**
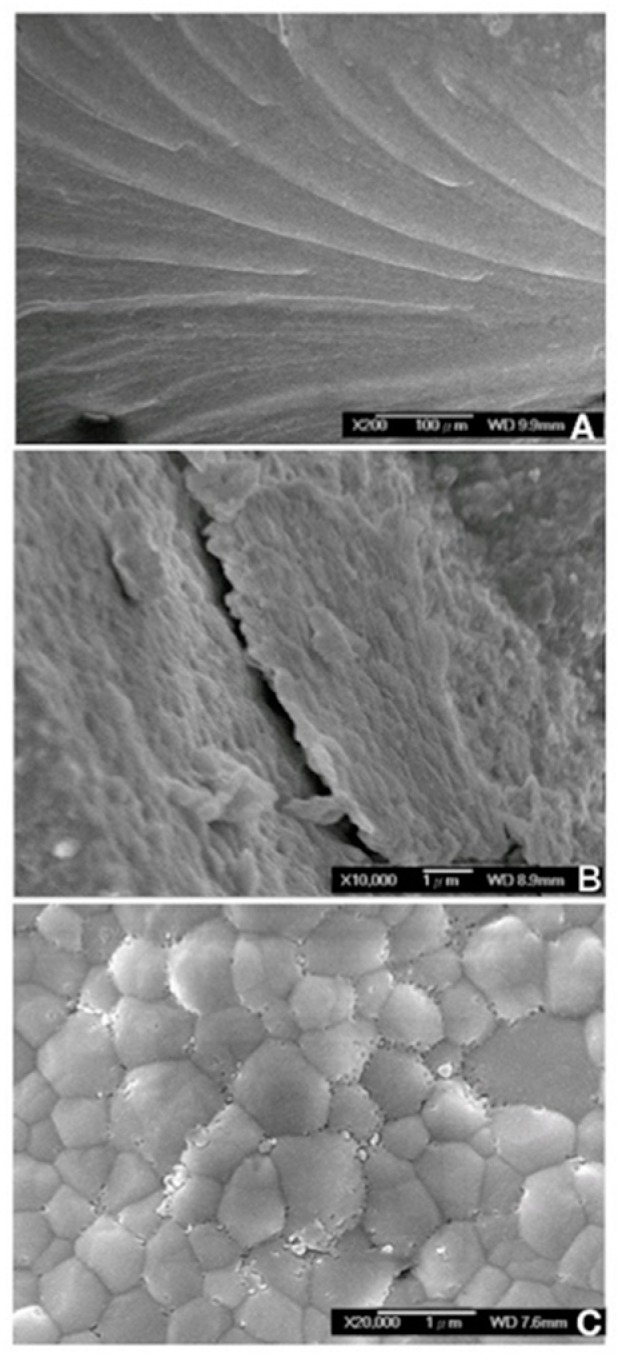
Scanning electron microscope images showing fracture conditions from broken segments. (**A**) Beach marks from the 0.4 mm fracture segments (original magnification, 200×, 100 μm, WD 9.9 mm). (**B**) Cracks observed from the 0.5 mm fracture segments (original magnification, 10,000×, 1 μm, WD 8.9 mm). (**C**) Intact surface of the zirconia specimens (original magnification, 20,000×, 1 μm, 7.6 mm).

Figure 7 shows the SEM microstructure of the fractured zirconia segment after loading. Figure 7A shows beach marks that indicate the progressive fatigue failure of the zirconia specimen. The final fracture line was visible alongside the beach marks when a brittle failure of the material occurred (Figure 7B). Figure 7C revealed the full densification and sufficient sintering of the zirconia occurred, and some small pores existed at grain and grain boundaries.

## 4. Discussion

Lawn et al. [39,40] demonstrated a linear relationship between ceramic specimen critical thickness and critical load. It showed that ceramic layer thickness much lower than 1.0 mm will most likely result in radial cracking from the lower surface. The present study revealed that the fracture strength of a zirconia specimen increased with thickness. Two of seven specimens with a thickness of 0.9 mm and all specimens with a thickness of 1.0 mm maintained an intact visible surface after 800 N cyclic loading. These results reject the null hypothesis and cause reasonable speculation that a thickness of 0.9 mm would be a turning point of fracture resistance in bruxism.

The FEM showed that the trend of peak stress value decreased with increased thickness. The 1.0-mm thick model showed the lowest peak stress values for all loading types. Crowns with a thickness of 0.9 mm showed a similar trend except for the oblique L (lingual) loading. The 0.4-mm thick model showed the lowest fracture strength and relatively higher peak EQV compared with the other thicknesses. Among the four oblique loading conditions, the peak value of EQVs of the crown from the lingual side was lower than the other side. This difference is attributed to the contact area of the prosthesis, with the radius of the contact area from the lingual side being greater than the others. The radius of the contact area negatively affected the stress under constant loading. Lan et al. [23] showed that thicknesses exceeding 0.7 mm had a high fracture resistance and lowest stress values under 300, 500, and 800 N loading. The present study showed a similar trend even with a different set of testing machines and FEM loading type.

Grinding and clenching are the most popular parafunction behaviors of bruxism. Tooth surface loss from bruxism might be divided into only functional cusp loss, called vertical bruxism, and full cusp loss, called horizontal bruxism [41]. Clenching always comes along with a large occlusal force and sometimes has signs of tooth wear, such as vertical, horizontal, or combined tooth surface loss. Grinding makes an obvious noise when sleeping, and it might affect tooth surface loss in a horizontal or oblique direction depending on the different grinding level. To place a posterior dental prosthesis in a patient with a history of bruxism, a clinician should consider the restorative materials and occlusion. Figure 5 shows the peak EQVs for four loading types, V 800 N, V 800 N + T 10 N, V 800 N + T 50 N, and V 800 N + T 100 N, on different thickness (0.4–1.0 mm). When the loading was simply from the vertical direction, it simulated clenching, and peak stress decreased with increased thickness. When the loading combined vertical force and torque, it simulated clenching and grinding. The peak value from V 800 N + T 100 N was higher than vertical force alone when the thickness was greater than 0.5 mm. The 1.0-mm thickness would be beneficial for use in clinical posterior implant zirconia prostheses in patients with bruxism due to the lowest peak EQV in all directions.

Clenching occurs by repetitive jaw-muscle activity with large loading, and grinding could expand the wear area of dentition. First, to reduce stress, the patient would be recommended to consult a neurologist, psychiatrist, or other physician. Second, after confirming cooperation from the medical field, the dentist should consider the biomechanical and biomaterial aspects of the treatment. It might always do more with less from the other specialist’s diagnosis and assistance. Third, fully densification and sufficient sintering of the zirconia was selected to construct the strength and long-term service life. Finally, correct interdental space calculation and routine follow-up would result in a beneficial situation.

Stress distribution should be a critical factor with bruxism. When the antagonist is a natural tooth, shock absorption is mainly provided by the periodontal ligament and partially by other organs, such as the alveolar bone. During clinical examination, a plausible technique is having light prosthetic contact (30 μm) when the occlusion record is heavy, while having no contact when occlusion record is light [13]. The new zirconia prosthesis would follow the wearing pattern of the antagonist and avoid premature and small contact areas to reduce peak stress concentration. Zirconia thickness would be suggested to be at least 1.0 mm, as proven by the present study. When the antagonist is an implant-prosthesis, the guidelines for implant occlusion in patients with bilateral free-end or who are completely edentulous are that there must be contact in MICP whether there is light or maximum intensity. The stress buffer would be provided only by the alveolar bone. The new prosthesis would allow for a less steep cusp inclination, wide cusp contact, and a small occlusal table on a wide implant body. The occlusal splint is recommended for daily using in these two conditions. Additionally, periodic active follow-up is needed, and radiographic and clinical observations and comparisons are imperative.

## 5. Conclusions

The fracture resistance of different thickness of zirconia by low-temperature sintering for dental use were studied using a servohydraulic testing machine, the FEM, and SEM. The fracture resistance revealed whether the specimen had broken or not (0.4–0.8 mm: all specimens had broken; 0.9 mm: two out of seven specimens were intact; 1.0 mm: all specimens were intact). The finite element analysis showed the 1.0-mm thickness specimens had the lowest peak EQV with different loading directions. Moreover, the zirconia followed the sintering temperature with proper densification of grain to bear long-term load from the microstructure findings.

## Figures and Tables

**Table 1:** Results of different occlusal thicknesses of zirconia specimens after cycling test under vertical 800 N loading.

Thickness (mm)	Total Cycle Number Mean ± SD	Specimen Number (n) Broken (+) or not (−)
0.4	5.4 ± 4.6 ^a^	7 (+)
0.5	12.4 ± 5.1 ^a^	7 (+)
0.6	27.1 ± 16.1 ^a^	7 (+)
0.7	1869.3 ± 2227 ^a^	7 (+)
0.8	10,346.9 ± 11,239.3 ^a^	7 (+)
0.9	50,853.4 ± 29,037.0 ^b^	5 (+)/2 (−)
1.0	100,000 ^c^	7 (−)
*p* value *	<0.01	<0.01

Different superscript letters in a column indicate statistical significance among groups (*p* < 0.05; post hoc Turkey test); 100,000 cycles stand for total number of cycles that specimens not broken (−) under vertical loading set. Specimen number (n) broken (+) means n specimens broken under 800 N loading. * Kruskal–Wallis test (K independent sample). Spearman’s correlation coefficients for zirconia specimen broken or not with different thicknesses (*r* = 0.659); Spearman’s correlation coefficients for total cyclic number with different thicknesses (*r* = 0.966). SD: standard deviation. ^a,b,c^ mean among class variation.
